# Recent Developments in Peptidyl Diaryl Phoshonates as Inhibitors and Activity-Based Probes for Serine Proteases

**DOI:** 10.3390/ph12020086

**Published:** 2019-06-10

**Authors:** Marta Maślanka, Artur Mucha

**Affiliations:** Department of Bioorganic Chemistry, Wrocław University of Science and Technology, Wybrzeże Wyspiańskiego 27, 50-370 Wrocław, Poland; 217458@student.pwr.edu.pl

**Keywords:** phosphonate esters, phosphorus peptide analogs, covalent inhibitors, enzyme activity imaging

## Abstract

This review presents current achievements in peptidyl diaryl phosphonates as covalent, specific mechanism-based inhibitors of serine proteases. Along three decades diaryl phosphonates have emerged as invaluable tools in fundamental and applicative studies involving these hydrolases. Such an impact has been promoted by advantageous features that characterize the phosphonate compounds and their use. First, the synthesis is versatile and allows comprehensive structural modification and diversification. Accordingly, reactivity and specificity of these bioactive molecules can be easily controlled by appropriate adjustments of the side chains and the leaving groups. Secondly, the phosphonates target exclusively serine proteases and leave other oxygen and sulfur nucleophiles intact. Synthetic accessibility, lack of toxicity, and promising pharmacokinetic properties make them good drug candidates. In consequence, the utility of peptidyl diaryl phosphonates continuously increases and involves novel enzymatic targets and innovative aspects of application. For example, conjugation of the structures of specific inhibitors with reporter groups has become a convenient approach to construct activity-based molecular probes capable of monitoring location and distribution of serine proteases.

## 1. Introduction

**Scheme 1:**
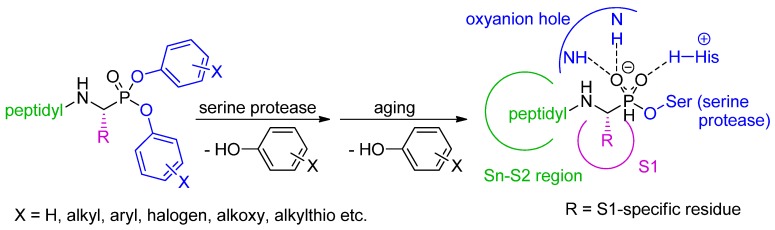
Structural features of peptidyl diaryl phosphonate inhibitors of serine proteases and the general mechanism of enzyme inactivation by transesterification with the active site serine hydroxyl functionality.

Diaryl α-aminoalkylphosphonates and their peptidyl extensions (in short, peptidyl diaryl phosphonates) are well-recognized, potent and selective mechanism-based inhibitors of serine proteases [1]. Because of the steric and electronic resemblance of the phosphorus moiety to substrates in the transition state of peptide bond hydrolysis, diaryl phosphonate esters are classified as transition state analogues. However, their mode of action is more complex than that exhibited by other phosphorus-containing pseudopeptides, and involves irreversible transesterification with the hydroxyl group of the active site serine, and formation of a covalent enzyme–inhibitor bonding which is accompanied by release of a phenol molecule [2]. Upon aging, the second phenol residue is also hydrolyzed to produce the final form of phosphorylated and thus inactivated enzyme (Scheme 1). Diaryl phosphonates do not inhibit proteases sharing similar modes of catalysis (cysteine, threonine), nor react with low-molecular nucleophiles, therefore they are stable in physiological media and nontoxic. Accordingly, they are used to study function and distribution of serine proteases, frequently those important in the context of human health [1,3]. The achievements in preparation of the active compounds over the period of last 6–7 years, together with their medical and diagnostic potential, are outlined in this paper.

## 2. Synthetic and Mechanistic Considerations

The potency, selectivity and specificity of peptidyl diaryl phosphonates can be conveniently regulated by three complementary adjustments of their structural features (Scheme 1) [3]. Fundamentally, the structure and configuration of the P1 side-chain substituent should fit to preferences of the S1 binding pocket of the targeted enzyme. At an early stage of phosphonate development, it was stated that for effective binding the absolute configuration of the α-carbon of the aminophoshonate fragment is preferentially (*R*) what corresponds to the (*S*) configuration of l-amino acid counterparts. Oleksyszyn and Powers used ^31^P NMR to show that one diastereomer of a peptidyl diphenyl phosphonate reacted faster [2]. This was confirmed by resolving of the crystal structures of serine protease–phosphonate complexes, e.g., [4], and obtaining enantiomeric phosphonates that show higher activity of the (*R*) configuration [5]. Secondly, the structure and reactivity of the leaving groups can be modified by appropriate substitution of the phenyl rings. These modifications modulate the electrophilic properties of the phosphorus atom by the electron withdrawing/donating effects [6]. As the substitution influences also specificity of the inhibitors, it is assumed that added groups or functionalities, e.g., *p*-SMe, improve contacts within the active sites upon initial binding [7]. Finally, to tighten interactions within the Sn-S2 region, the N-terminus of the basic aminophosphonate structure can be elongated by amino acids or peptides to provide extended derivatives. These diastereomeric peptidyl products are easily separated chromatographically. The (*R*) configuration of the aminophosphonate portion is typically assigned to the epimer that is more reactive with its enzymatic target. The assignment can be further supported by molecular docking and the chemical shift of ^31^P NMR resonances (for exemplified clarification see [8,9]).

Formation of the covalent enzyme–phosphonate complex was found to be slowly reversible [2]. The phosphonate cleavage could be visibly accelerated in the presence of specific reagents; thus, the activity of a serine protease is restored. Recently, Ono et al. studied pyridinium oxime 2-pyridine aldoxime methiodide, an effective acetylcholinesterase reactivator used as an antidote against nerve agent poisoning, to reactivate chymotrypsin after transesterification with diphenyl phosphonate derivatives [10,11,12]. Dephosphonylation readily occurred prior to aging of the complex and was applied for enzyme purification and labeling. Gly_3_-PheP(OPh)_2_ (Gly-Gly-Gly-Phe tetrapeptide phosphonate analog) immobilized on a Sepharose gel, served to capture chymotrypsin-like proteases in covalent chromatography [10]. The highly purified enzymes were then released under the action of the oxime in a rapid and versatile procedure.

**Scheme 2:**
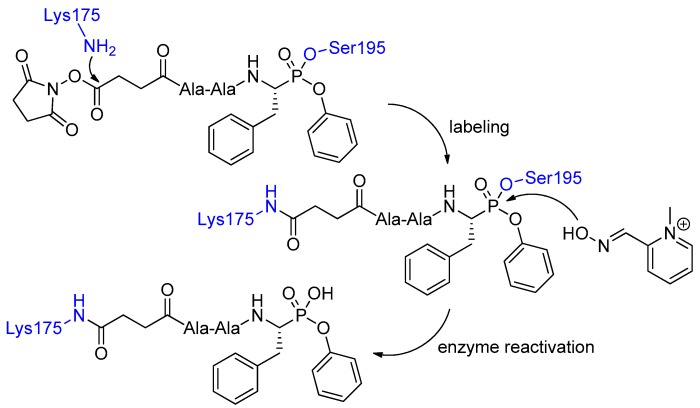
Selective labeling of Lys175 of chymotrypsin with Ala-Ala-PheP(OPh)_2_ proceeding via enzyme phosphonylation and reactivation induced by the oxime reagent.

In continuation, bifunctional (containing additional reactive *O*-succinimide ester group) peptidyl phosphonates were found to modify effectively Lys175 in chymotrypsin [11]. The whole binding-and-release cascade involved Ser195 transesterification, subsequent conjugation of *O*-succinimide ester with the Lys175 residue and ultimate pyridinium oxime 2-pyridine aldoxime methiodide-mediated cleavage of the P-terminus (Scheme 2). Bioconjugation proceeded stereoselectively as evidenced by application of diastereoisomers that were resolved chromatographically [11]. The lll-configured epimer reacted much more readily than the lld-configured one. To explain these observations the authors speculated that binding of the more reactive stereoisomer gave the covalent complex fixed in a favorable orientation, stabilized by interactions similar to those formed by a substrate of natural configuration [12]. Consequently, orientation in the oxyanion hole and the proximity of His57 with its base–acid catalytic properties facilitated not only P–O bond formation and breaking, but also the reaction at the N-terminus.

**Scheme 3:**
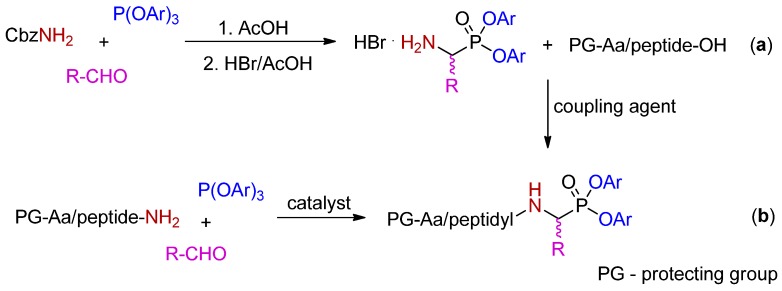
Methods for the synthesis of oligopeptidyl diaryl phosponates: the approach based on the three-component Birum–Oleksyszyn condensation followed by one-step incorporation of a peptide fragment (pathway **a**), an alternative version with the reverse order of the reactions (pathway **b**).

Oligopeptidyl diaryl phosphonates are currently of major interest for their improved recognition by the Sn-S2 binding sites of targeted hydrolases. The classical approach to obtain such derivatives involves synthesis of diaryl α-(*N*-benzyloxycarbonyl)aminoalkylphosphonates in the three-component Birum–Oleksyszyn condensation of triaryl phosphite, benzyl carbamate and an aldehyde [13], *N*-deprotection of the product, and chain extension with amino acid or peptides, with the use of typical activators, such as: EDC/HOBt, HBTU, HATU etc. The couplings are required to be performed in solution and for oligopeptide derivatives are more effective when accomplished in a single step. For example, the total yields for the synthesis of Ac-Val-Phe-Leu-Leu-LeuP(OPh)_2_ increased by five-fold from less than 5% for sequential step-by-step amino acid incorporation to 25% for coupling of the tetrapeptide (presynthesized on the solid phase) with H-LeuP(OPh)_2_ (Scheme 3, pathway **a**) [14]. The approach based on the ultimate P–C-forming amidoalkylation of the whole sequence peptidyl amide with triaryl phosphite and an aldehyde gave comparable yield (26%) (Scheme 3, pathway **b**). In this method copper(II) triflate was used as a Lewis acid catalyst in milder conditions than those demanded in the case of the acetic acid-mediated condensation [14]. 

**Scheme 4:**
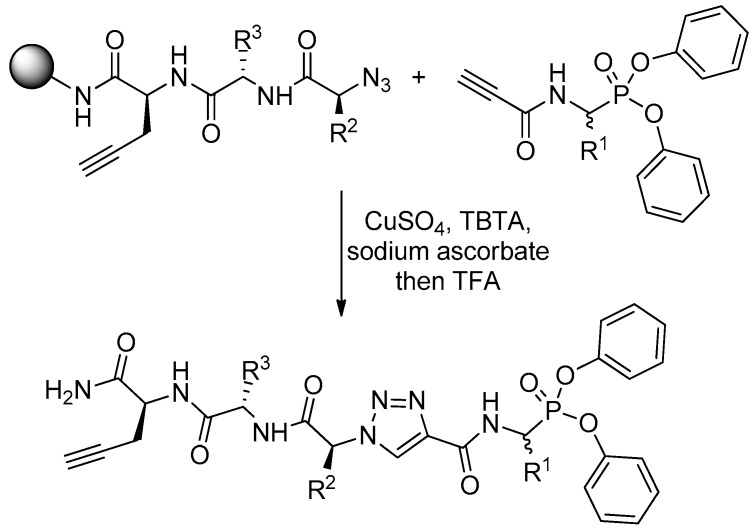
Solid phase “click” methodology for the synthesis of triazine activity-based probes, established by linking a diphenyl phosphonate warhead with an oligopeptide fragment.

Diaryl α-aminoalkylphosphonates are hardly adapted to the classical C-terminus-immobilized solid phase peptide synthesis. To perform this, one needs to change “the direction” of elongation in a certain sequence point. In Verhelst groups such an approach, based on “click” chemistry, was suggested to explore the selectivity of diphenyl phosphonate activity-based probes for serine proteases [15]. The N-termini of solid phase-immobilized specific tripeptides were transformed into azide by triflyl azide and then clicked with diphenyl *N*-acetylenecarbamidoalkylphosphonates (Scheme 4). Thus, the C- and P-termini were linked together by central triazine system. The phosphonate worked as a warhead while C-terminal propargylglycine residue enabled visualization, also by “click” chemistry with a dye azide, after labeling of a target protease.

## 3. Inhibition of Serine Proteases

Two main directions can be distinguished in the current research on biological activity of peptidyl diaryl phosphonates. A quest for more potent and selective inhibitors that could control the action of serine protease in the therapeutic context is the first one. Secondly, new covalent ligands are studied as convenient tools to monitor the role, function and distribution of these enzymes as activity-based probes. The most comprehensive recent studies on these issues concern elaboration of active site-directed diaryl phosphonates targeted to serine protease secreted by neutrophils. Being a part of the innate immune system neutrophils release hydrolases, e.g., cathepsin G, neutrophil elastase and proteinase 3, that are associated with a response to invading pathogens [16]. Because of the roles in host defense and disease, they are of interest as potential therapeutic targets [17].

Studies on inhibition of human neutrophil elastase by peptidyl phosphonates provided extremely reactive and specific compounds. The enzyme under physiological conditions is dedicated to defense against bacterial infection but it also has an ability to cleave the majority of extracellular matrix components and activate metalloproteinases, therefore its activity is rigorously endogenously controlled by serpin inhibitors. Upregulation of human neutrophil elastase activity causes chronic obstructive pulmonary disease and other pulmonary respiratory syndromes. Improving the potency of Cbz-ValP(OPh)_2_ against the elastase, Winiarski et al. designed a series of tripeptide analogs bearing the optimized P2 and P3 residues and the leaving groups [8]. Furthermore, to overcome hydrophobicity of the sequence, the *N*-termini of active structures were modified with heteroatom-rich hydroquinone, thymine or uracil derivatives. As the result, the most potent inhibitors of human neutrophil elastase, soluble in aqueous media and selective versus porcine pancreatic elastase, chymotrypsin and trypsin, were obtained (Table 1, Entry **1**).

Peptidyl di(*p*-chlorophenyl) phosphonates enabled selective inhibition of human neutrophil proteinase 3, thus, its differentiation from the structural homolog, neutrophil elastase. Functions of neutrophil proteinase 3 are much poorly recognized than elastase and can be partially clarified by a small extent of exposition of the enzyme on the outer surface of circulating neutrophils, which could suggest a role as an autoantibody target in vasculitides, and its likely involvement in cell apoptosis [18]. The structure of the inhibitors was based on a privileged sequence obtained from studies on cleavage of FRET substrates. In particular, P2 aspartate and P4 proline residues were found favorable (Table 1, Entry **2**) [19]. The N-terminus of the compound was biotinylated to play a role of the reporter in activity based-probes. The subsequent kinetic studies, aided with molecular modelling, brought further optimization of the elaborated structure. Accordingly, the length of the P1 residue was extended, and P4 Pro was conveniently replaced with Val. These changes gave rise to an increase of the second-order inhibition constant by more than an order of magnitude (7 × 10^5^ M^−1^s^−1^) as compared to the lead tetrapeptide [20].

Two guanidinophenyl residues were applied as arginine mimetics in construction of dipeptide diaryl phosphonate analog inhibitors of matriptases [21,22]. Matriptase activates several substrates, which play critical roles in tumorigenesis, while matriptase-2 is a transmembrane serine protease involved in regulation of iron homeostasis though a signaling pathway. Matriptase-2 inhibits activation of systemic regulatory hormone hepcidin by cleaving membrane hemojuvelin, a phosphorylating coreceptor. The structure of the matriptase-2 inhibitors was supported with the sequence of a privileged substrate (Ile-Arg-Ala-Arg) and docking analysis [22]. The *para* position of the guanidine groups, the natural configuration (*S,R*), and *para*-(methylthio)phenoxy leaving groups appeared to be favorable structural features to produce the most reactive inhibitor (Table 1, Entry **3**). Two guanidinophenyl-based diaryl phosphonates were inactive towards human thrombin and bovine factor Xa, yet they inhibited bovine trypsin.

The nonproteinogenic secondary amino acid indoline-2-carboxylic acid (Table 1, Entry **4**) was used to develop a diaryl phosphonate inhibitor of tripeptidyl peptidase II, which shed light on unknown mechanism of the enzyme participation in various biological processes, among others, in cell growth, DNA repair and signaling. Irreversible action of *N*-(l-2-aminobutyryl)-l-indoline-2-carboxyl-LeuP(OPh)_2_ rapidly decreased the levels of active signal-regulated kinase 1 and 2 in the nucleus, thereby down-regulating signal transduction downstream of growth factors and mitogenic stimuli [23].

Serine proteases of human pathogens, which are indispensable for growth and virulence, are attractive drug targets [28,29]. These proteases have been also studied with peptidyl aryl phosphonates. For example, inhibition of the bifunctional activity (serine protease and NTPase/RNA helicase) of hepatitis C virus nonstructural protein 3, which is fundamental for viral polyprotein processing, RNA replication virion formation and blocking innate immune pathways [30], was recognized as an effective antiviral therapy [31]. Following this assumption Skoreński et al. constructed a potent irreversible phosphonate inhibitor of two genotypes and four mutants of NS3/4A protease (Table 1, Entry **5**) [24]. The most active structures contained, crucial for their potency, constrained bicyclic proline analogs, utilized previously to develop approved drugs Boceprevir (VICTRELIS™) [32] and Telaprevir (INCIVEK/INCIVIO™) [33]. Interestingly, the newly obtained compounds did not shown any cytotoxicity up to the concentration determined by their solubility in water (1–10 mM).

The West Nile virus serine protease NS2B/NS3 was also targeted with diaryl α-aminoalkylphosphonates by the same group [34]. A set of lysine/arginine mimetics were preliminarily evaluated to recognize the preferences of the S1 binding pocket. *para*-Guanidino substituted phenylglycine and phenylalanine analogs were found the most potent inhibitors. N-Termini elongation to Cbz-Lys-Arg-(4-guanidino)PheP(OPh)_2_ yielded a compound which displayed *K*_i_ = 0.4 µM and *k*_2_/*K*_i_ = 28,265 M^−1^s^−1^.

Extensive screening of diaryl α-aminoalkylphosphonates and their elongation to oligopeptide derivatives allowed finding inhibitors of serine protease infection factors of *Staphylococcus aureus*. A range of these proteases is indispensable for virulence to cleave extracellular components, distract host defense and immune system functioning, however, their specific roles are not fully recognized yet. Tripeptide phosphonate analog inhibitors of endoproteinase GluC (V8 protease), and SpIA and SpIB proteases are listed in Table 1 (Entries **6–8**). Their structures mostly evolved from enzyme substrate preferences at the P1 position followed by optimization of P2 and P3 residues and substitution of the aromatic leaving group. Accordingly, the Phe-Leu-Glu sequence appeared favorable for endoproteinase GluC (Table 1, Entry **6**), which was inhibited selectively versus SpIB protease [25]. In turn, the last-mentioned enzyme was inactivated by Glu-Leu-Gln phosphonate that demanded appropriate adjustment of structure and reactivity of the leaving phenyl group (Table 1, Entry **8**). 4-Methoxy substitution was found optimal, however, this compound was also potent towards subtilisin [27]. For SpIA protease, N-protected hydrophobic tripeptide derivatives (Val-Pro-Leu and Val-Pro-Phe, Table 1, Entry **7**) typically appeared more potent than nonextended α-aminoalkylphosphonates [26]. Selected inhibitors served for cocrystallization with this enzyme and provided high resolution structures of the ligand–protein complexes. Surprisingly, the evidenced binding mode was noncanonical as SpIA protease was phosphonylated with an epimer of non-natural (*S*)/d configuration at the P1 position, which was selected from the stereomeric mixture. The overall conformation of the tripeptide chain was also distorted. The chain was flexible and did not form typical antiparallel arrangement with the enzyme backbone in the Pn region [26].

Tripeptide analog Boc-Val-Pro-ValP(OPh)_2_ was recently reported to alter virulence of another species of pathogenic bacteria. Significant loss of *Chlamydia trachomatis* infectious progeny was observed when the cell culture was treated with 10 µM of the phosphonate in the mid-replicative phase [35]. Antichlamydial activity was measured to be 100-fold higher for the lll diastereoisomer compared to the epimer bearing the d-ValP(OPh)_2_ fragment. Plausible inactivation of *Chlamydia* high temperature requirement A protease, a multimeric and multidomain serine protease that is indispensable for the pathogen virulence, was postulated as the reason of the activity. This suggestion was indirectly proven by submicromolar inhibition of human neutrophil elastase, a protease of similar substrate specificity.

Lastly, screening of focused libraries of diaryl phosphonate-based serine protease inhibitors led to identification of several potent inactivators of the *Escherichia coli* caseinolytic protease subunit P (ClpP) [36]. ClpP is widely conserved in bacteria, modulates virulence factor expression and thus regulates virulence and stress response [37]. N-Cbz-protected diphenyl phosphonate analogs of phenylglycin, substituted in meta position with either amino or guanidino groups, appeared to be the most potent, with IC_50_ = 0.5 µM.

## 4. Activity-Based Probes

**Figure 1:**
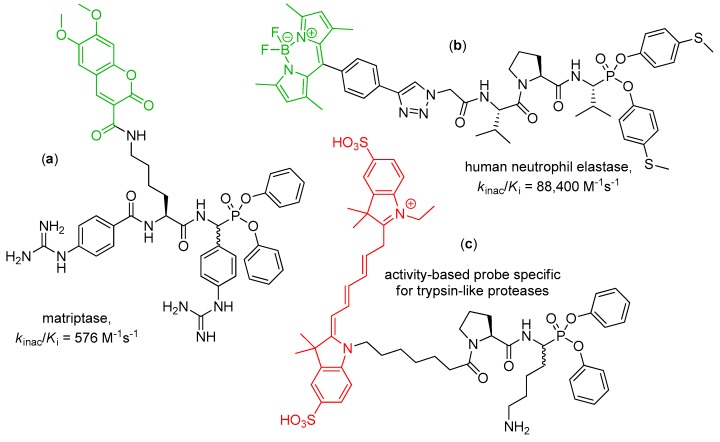
A selection of fluorescently labelled phosphonylating inhibitors for detection of serine protease: a coumarin-based probe for matriptase (**a**), a BODIPY probe for human neutrophil elastase (**b**), and a cyanine-modified peptidyl diphenyl phosphonate specific for trypsin-like proteases (**c**).

Peptidyl diaryl phosphonates are a perfect platform to develop activity-based probes, molecules that enable monitoring active forms of recombinant and native serine proteases, in analytical techniques and in vivo [38,39]. Phosphonate inhibitors fulfill essential demands for activity-based probes—they are irreversible and active site-oriented, moreover, their structure can be easily refined to achieve high reactivity and selective binding. The structure demands only decoration with a reporter fragment, typically at the *N*-terminus. Two main approaches to envisage a probe–protein complex are based on introduction of either an intrinsic fluorescent fragment or a moiety that can be fluorescently labelled/recognized in a subsequent step [40,41,42,43]. In fact, recent studies on diaryl phosphonate inhibitors of serine proteases are frequently accompanied by elaboration of fluorescent activity-based probes. Some examples of application of fluorophore-tagged phosphonates have been mentioned in the preceding chapter, e.g., the use of propargylglycine to be clicked with an azide rhodamine derivative for detection of chymotrypsin and related enzymes [15]. Gütschow and coworkers also modified the structure of a potent irreversible phosphonate inhibitor to provide the first activity-based probe of matriptase-2 [22]. The developed bisbenzguanidines chemotype contained 7-diethylaminocoumarin as a fluorescent dye. The coumarin acetaminomethyl reporter tag replaced the benzyl residue in the P2 position of the parent inhibitor molecule (for the structure see Table 1, Entry **3**). The probe allowed for direct matriptase-2 detection in a complex protein mixture separated by gel electrophoresis. In continuation, a similar coumarin-based probe of matriptase was developed by the corresponding design and synthesis approach (Figure 1, compound **a**) [44]. To label the inhibitor, 6,7-dimethoxycoumarin-3-carboxylic was coupled with ω-amino group of the P2 lysine residue. The utility of the final compound was evaluated by in-gel fluorescence and, for the first time for proteases, in fluorescence HPLC.

The same group developed a fluorescent activity-based probe for human leukocyte elastase on the basis of Val-Pro-ValP(OPh-*p*-SMe)_2_ inhibitor sequence and a boron-dipyrromethene (BODIPY) label (Figure 1, compound **b**) [9]. The fundamental phosphonate tripeptide was synthesized and resolved chromatographically to yield pure diastereoisomers. Subsequent incorporation of azidoacetic acid to the more reactive stereoisomer (*k*_inac_/*K*_i_ = 399,000 M^−1^s^−1^, 400-fold higher compared to the less reactive epimer) allowed for copper catalyzed 1,3-dipolar cycloaddition with the ethinyl group of a label. The probe maintained high potency against human leukocyte elastase and good selectivity versus porcine pancreatic elastase. SDS-PAGE and fluorescence analysis showed a selective elastase imaging.

Edgington-Mitchell et al. synthesized and characterized two activity-based probes containing Cy5, a near-infrared fluorophore suitable for in vivo imaging [45]. The cyanine building block was coupled either with ValP(OPh)_2_ to target elastase-like proteases or Pro-LysP(OPh)_2_ to target trypsin-like enzyme (Figure 1, compound **c**). The probes efficiently labelled purified protease, also in complex mixtures. With these tools an elevated level of trypsin-like proteases was evidenced in two models of inflammation. Low elastase activity suggested upregulation of endogenous inhibitors or degrading proteases.

**Figure 2:**
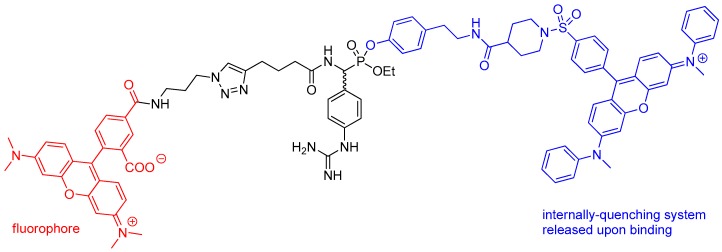
The structure of an internally-quenched fluorescent activity-based probe for labeling of arginine-specific serine proteases.

Mixed alkyl aryl phosphonate esters were designed as quenched fluorescent activity-based probes [46]. This type of probe contains a fluorescence quencher that is released upon binding as a part of a leaving group, which makes a free molecule appear dark while making an enzyme-bound one fluorescent. To obtain the compound, tetramethylrhodamine fluorophore was conjugated by the azide group with a triple bond of presynthesized phosphonate system by “click” chemistry, similarly to the method shown in Scheme 4. The product (Figure 2) was evaluated for labeling of trypsin, urokinase plasminogen activator, cathepsin G, chymotrypsin and elastases.

**Figure 3:**
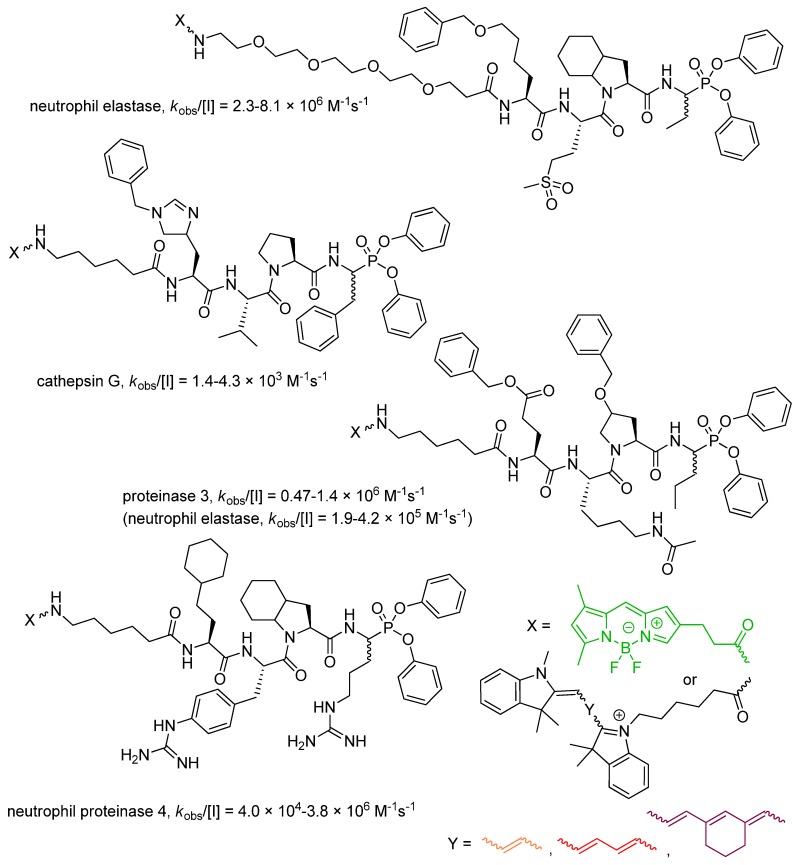
A unique toolbox of fluorescent diaryl phosphonate probes for parallel imaging of human neutrophil serine proteases. Specific sequences and their activities, together with the structures of the reporter fluorophores, are presented.

Drag and Salvesen laboratories developed a general toolbox of fluorescent diaryl phosphonate probes for parallel imaging of all four active neutrophil serine proteases [47]. To ensure a high selectivity ratio, the optimized P1–P4 recognition sequences were established by using mostly nonproteinogenic amino acids (Figure 3) and the Hybrid Combinatorial Substrate Library (HyCoSuL) [48]. For elastase and proteinase 3 the probes appeared highly efficient, while for cathepsin G and proteinase 4 the activity was less striking, nevertheless, a high discrimination between human neutrophil serine proteases was achieved. The only cross reactivity was observed for proteinase 3 inhibitor with elastase. The intelligent application of fluorescent reporter groups with minimal wavelength overlap allowed for simultaneous observation and detection of all individual proteases by fluorescence microscopy. A non-overlapping distribution of four different neutrophil serine proteases in the azurophil granules was unprecedentedly demonstrated [47].

**Figure 4:**
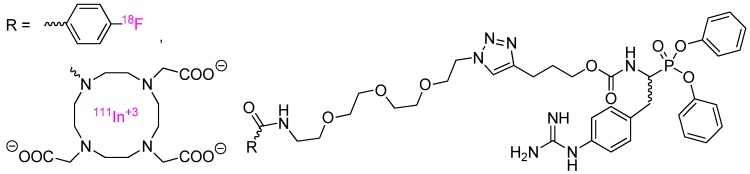
Radiolabelled activity-based probes for imaging in positron emission tomography or single photon emission computed tomography, based on the structure of *p*-guanidino-PheP(OPh)_2_, an inhibitor of urokinase-type plasminogen activator.

Urokinase-type plasminogen activator which facilitates tumor cell invasion and metastasis by the degradation of the basement membrane and the extracellular matrix was targeted with radiolabelled activity-based probes and positron emission tomography [49] or single photon emission computed tomography imaging [50]. The synthesis of probes involved coupling of specific inhibitor *p*-guanidino-PheP(OPh)_2_ with either [^18^F]-4-fluorobenzoyl group or a DOTA chelator using the “click” chemistry methods and a polyethylene glycol linker (the structures depicted in Figure 4).

Subsequently, the tetraza macrocycle was complexed with ^111^In radioisotope. The fluorine compound was found to be slightly more potent inhibitor of human urokinase (*k*_app_ = 6800 M^−1^s^−1^, IC_50_ = 19 nM) than the DOTA complex (*k*_app_ = 4800 M^−1^s^−1^, IC_50_ = 22 nM), however, it showed low tumor uptake because of unfavorable stability and poor pharmacokinetic properties. Moderate uptake of indium complex was found in two tumor models as clearly detected in single photon emission tomography 4 days post injection.

**Figure 5:**
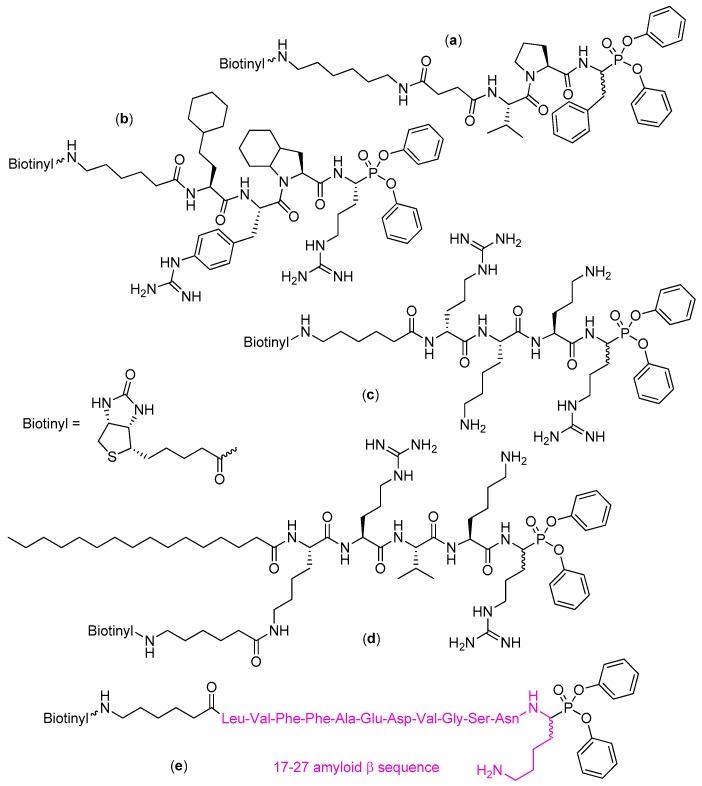
Highly potent and specific activity-based-probes with the diphenyl phosphonate warhead and biotin reporter for detection of serine protease activity of cathepsin G (**a**), human neutrophil serine protease 4 (**b**), the structure developed with the use of HyCoSuL methodology), the Zika virus NS2B-NS3 protease (**c**), furin (**d**), and amyloid β peptide-hydrolyzing antibodies (**e**).

N-Biotinylation of the inhibitors and recognition with an extravidin/streptavidin system is an alternative indirect method for fluorescent visualization of serine proteases [42]. This approach has been mentioned in the preceding chapter for the development of biotinyl-Val-Tyr-Asp-nValP(OPh-*p*-Cl)_2_, a probe for the human neutrophil proteinase 3 [19,20]. In continuation, biotin conjugated with Val-Pro-Phe phosphonate via an appropriate linker yielded also a highly sensitive probe for detection of cathepsin G in western blot and 96-well-based high-throughput assays (Figure 5, compound **a**) [51]. This and other biotin-modified inhibitors were also used to study inhibition and detection of neutrophil serine proteases in spleen lysates [52]. In kinetic test they were found potent and selective, e.g., phosphonate **a** depicted in Figure 5 reacted with cathepsin G (*k*_obs_/[I] = 3800 M^−1^s^−1^), but not with human neutrophil elastase, nor proteinase 3. In a contrary, *N*-biotinyl-Val-Pro-ValP(OPhe-*p*-MeS)_2_ inactivated elastase with *k*_obs_/[I] = 550,000 M^−1^s^−1^, proteinase 3 with *k*_obs_/[I] = 16,000 M^−1^s^−1^, while it was inactive toward cathepsin G [52]. The high activity corresponded to the low limits of detection, for example nanograms and even lower amount of cathepsin G could be visualized by western blotting with the use of the most potent probes what outscored immunostaining with specific antibodies. The active enzyme could be also simply detected in biological samples as tested on peripheral blood mononuclear cells (accumulation on the cell surface) and spleen extracts. In a complementary study biotinyl-[PEG]_66_-Pro-Tyr-Asp-AlaP(*p*-Cl-Ph)_2_, an extended version of inhibitor of neutrophil protease 3 depicted in Table 1 (Entry **2**), served to show distribution of the enzyme in permeabilized neutrophils [19]. The cytoplasm was intensively labeled but not the perinuclear environment as observed for human neutrophil elastase.

Kasperkiewicz et al. [53] reported a very potent, active and selective biotin-based probe for human neutrophil serine protease 4, the newly discovered fourth member of the neutrophil serine proteases family [54]. To obtain a privileged sequence that would match with the subsite preferences, the authors screened a range of natural and unnatural amino acids. Identification of the optimal substrate sequence was followed by its translation into the inhibitor structure (Figure 5, compound **b**). The more active diastereoisomer (presumably of the (*R*)/l configuration at the P1) displayed 40-fold higher potency (*k*_obs_/[I] = 3,800,000 M^−1^s^−1^) than its less potent epimer.

An excellent diphenyl phosphonate activity-based probe for the Zika virus NS2B-NS3 protease, based on tetrapeptide d-Arg-Lys-Orn-Arg motif, was also constructed (Figure 5, compound **c**) [55]. Apparent second-order rate constant for inhibition under pseudo first-order conditions was measured at 2,940,000 M^−1^s^−1^. The compound bound immediately to the protease which allowed effective labeling in one minute of incubation.

Construction of a furin inhibitor, palmitoyl-Lys(*N*-biotinyl-6-aminohexanoyl)-Arg-Val-Lys-ArgP(OPh)_2_, was also based on the specific substrate core which was equipped with C-terminal reactive warhead and N-terminal biotin reporter fragment for visualization (Figure 5, compound **d**) [56]. Furin is a serine protease, the member of mammalian proprotein convertases, which may activate viral and bacterial pathogenic proteins in addition to its physiological role of trimming a number of enzymes, hormones, signaling molecules, transcription and growth factors to their active forms [57]. The compound inhibited the enzyme with *K*_i_ = 0.93 µM and *k*_3_/*K*_i_ = 20,500 M^−1^min^−1^ with a great selectivity as it appeared inactive towards chymotrypsin, trypsin-like proteases and neutrophil serine proteases up to 10 μM concentration. This contrasted to the behavior of the reference chloromethyketone of the corresponding consensus sequence.

The electrophilic diphenyl phosphonate warhead served to design and develop an activity-based probe for antibodies capable of hydrolysis of amyloid β peptide by serine protease-like mechanism [58]. The eleven amino acid long sequence was determined by the lysine-specific cleavage sites (Figure 5, compound **e**). As evidenced by electrophoresis and western blotting the probe was specifically and covalently bound to the antibodies to form a stable complex. It also reacted with the target proteins in serum, thus, allowing effective identification and isolation of amyloid β peptide-hydrolyzing antibodies.

## 5. Conclusions

Peptidyl diaryl phosphonates have been validated as an expedient framework for the development of potent and selective inhibitors of serine proteases. These inhibitors exhibit not only potency but also favorable physicochemical and pharmacokinetic properties. The ester phosphonate functionality ensures their resistance to unspecific proteolysis and good stability in buffer and plasma. They reveal specific reactivity in an exactly targeted location, including intracellular destinations. Thus, several diaryl phosphonate compounds of optimized structure have been investigated as drug candidates against proteases involved in the development of human diseases, such as urokinase-type plasminogen activator which facilitates tumor cell invasion and metastasis by the degradation of the basement membrane and the extracellular matrix, or dipeptidyl peptidase IV, of which inhibition stimulates insulin secretion and reduces the blood glucose level in the treatment of type 2 diabetes [3,59,60]. Following these achievements we have presented the current state of development in this area. First, the utility of diaryl phosphonate has been confirmed for viral and microbial serine proteases, the enzymes indispensable for growth or virulence of human pathogens. As a consequence these proteases and their inhibitors have been pointed out as molecular targets and specific drugs of antibacterial/antiviral activity. Secondly, peptidyl diaryl phosphonates conjugated with a suitable reporter group are extensively explored for intelligent detection of the enzymes in vitro and in vivo in simple and highly sensitive tests.

## Figures and Tables

**Table 1:** A selection of recent achievements in the development of peptidyl diaryl phosphonate inhibitors of serine proteases.

Entry	Inhibitor Structure	Targeted Enzyme, Activity and Reference
**1**	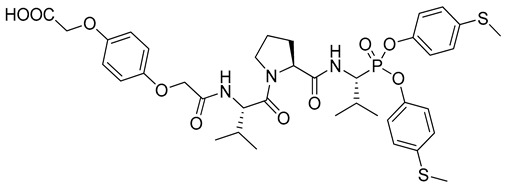	Human neutrophil elastase, *k*_inact_/*K*_I_ = 2,353,000 M^−1^s^−1^ [8]
**2**	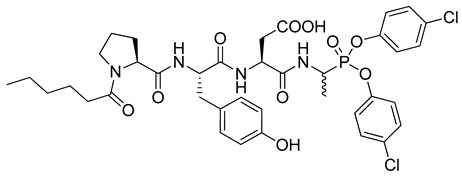	Human neutrophil protease 3, *k*_obs_/[I] = 4418 M^−1^s^−1^ [19],
**3**	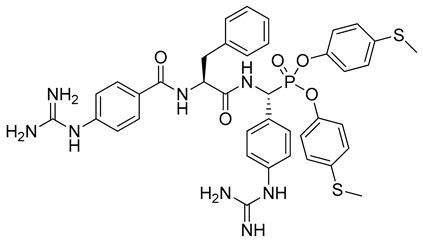	Human matriptase-2 *k*_inact_/*K*_i_ = 2790 M^−1^s^−1^ [22]
**4**	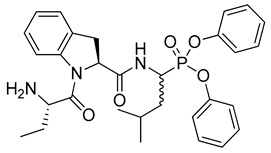	Tripeptidyl peptidase II, IC_50_ = 0.46 nM [23]
**5**	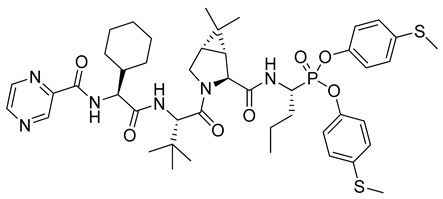	Hepatitis C virus NS3/4A protease, *k*_2_/*K*_i_ = 79,850 M^−1^s^−1^ (genotype A), *k*_2_/*K*_i_ = 60,850 M^−1^s^−1^ (genotype B) [24]
**6**	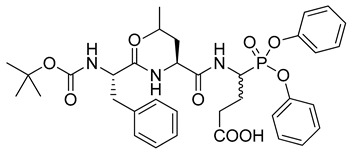	*Staphylococcus aureus *endoproteinase GluC *k*_2_/*K*_i_ = 8540 M^−1^s^−1^ [25]
**7**	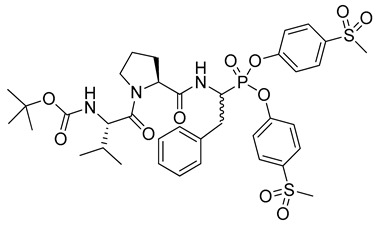	*Staphylococcus aureus* SpIA protease *k*_2_/*K*_i_ > 8000 M^−1^s^−1^ [26]
**8**	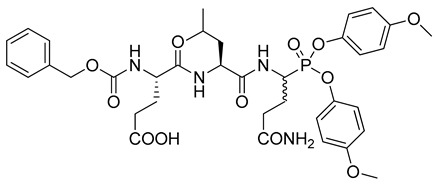	*Staphylococcus aureus* SpIB protease *k*_2_/*K*_i_ = 1400 M^−1^s^−1^ [27]
